# Supplementary material for: Chromothripsis during telomere crisis is independent of NHEJ, and consistent with a replicative origin
Source: Genome Res. 2019 May;29(5):737–49. doi: 10.1101/gr.240705.118 (PMC6499312; doi:10.1101/gr.240705.118)
Supplement: Supplemental Material [file supp_gr.240705.118_Supplemental_file_1.zip › contigs/annotated_contigs/DB80/contig.2.DB80_length_650_mean_cov_11.5107692308.docx]

**DB80_length_650_mean_cov_11.5107692308**

```
CA|AAATTAGGTAAAGTGTCACCAAGTGAATGTGTGTGTGTTAGTCCAGGTCTTGGAATTGGGTTTACCATAATTGTAGGCCAAGTATT
   >chr18:23003680-23004032 - E=4e-200
CAGGACAATAAACAGCCTCAGGGGCACAGGTCTTGAGAGTTCAAACCTATAAACCTTAAAGAGATGGAAAATATTACTCCTAGGAGGCT

TACTGTGGTTTTTTGTTCCATCAAAGCAAATTACACAGAGACATCATGAATTGATGCAAATTCAGTCCAGTCTCTAGTGCTGCTACTCA

ATAACATGAACTATACTTGCATTTCTCATTTTTCTTTTTTACTTCTGTGATCCTCCTTTTTGTCATTTTGTGGTTTATTTCTACT|TCT
                                                                                      >c
|CTCATGAACTGCTAGGTAAATTGGGTGAAAGGGCTTCAGTTCCTTGATAAAGTATAGAATAAACATTTCATTGATAGACCAAAGGGTT
hr18:23002504-23002803 - E=4e-168
TAGAAAAACAACTGTAAATTGTCCAATATAGCATCCAAGCAATTTAGCATGGTGTGAAAGTCAAGAATTAGCACCTCCAATAGGTGAAT

TTGAGTTTCATCTTTTCCCCCTGTTTTTGCCCACCCCTGTGGGAGGTGATAATGAATAGTGAAATGGTCAAGTTAGGTCTCAACAGGAG

GGAGAGGGAAGGGTGGCCAATGCCTTCAAA
```
